# CT-based radiomics and deep learning to predict EGFR mutation status in lung adenocarcinoma

**DOI:** 10.3389/fonc.2025.1597548

**Published:** 2025-10-02

**Authors:** Xingzhi Jiang, Qian Sun, Can Wang, Wei Li, Wang Chen, Juan Xu, Lei Yu

**Affiliations:** ^1^ Department of Respiratory Medicine, the First People’s Hospital of Yancheng, Yancheng, China; ^2^ The Yancheng Clinical College of Xuzhou Medical University, Yancheng, China; ^3^ Department of Pulmonary and Critical Care Medicine, Zhongda Hospital, Medical School, Southeast University, Nanjing, Jiangsu, China; ^4^ Department of Radiology, the First People’s Hospital of Yancheng, Yancheng, China; ^5^ Yancheng No.1 People’s Hospital, Affiliated Hospital of Medical School, Nanjing University, Yancheng, China

**Keywords:** EGFR, CT, deep learning, radiomics, fusion model, lung adenocarcinoma

## Abstract

**Objectives:**

Epidermal growth factor receptor (EGFR) mutation status is an essential biomarker guiding targeted therapy selection in lung adenocarcinoma. This study aimed to develop and validate a non-invasive predictive model that integrates radiomics and deep learning using CT images for accurate assessment of EGFR mutation status.

**Methods:**

A total of 220 patients with lung adenocarcinoma were retrospectively enrolled and randomly divided into training and testing cohorts at a 7:3 ratio. Radiomics features were extracted from CT images using PyRadiomics, and deep learning features were obtained from five pretrained architectures: ResNet34, ResNet152, DenseNet121, ShuffleNet, and Vision Transformer (ViT). Feature selection used the intraclass correlation coefficient, Spearman correlation, and LASSO regression. The deep learning architectures were compared within the training set using cross-validation, and the best-performing architecture, ViT, was retained for downstream modeling. Based on the selected features, we constructed a radiomics model (Rad model), a ViT-based deep learning model (ViT model), and two fusion models (early fusion and late fusion) integrating radiomics and ViT features. Model performance was evaluated using receiver operating characteristic (ROC) curves, area under the curve (AUC), accuracy, sensitivity, specificity, precision, F1-score, and decision curve analysis (DCA).

**Results:**

The fusion models outperformed both radiomics and deep learning models in predicting EGFR mutation status. In the testing set, the early fusion model achieved the highest predictive performance (AUC = 0.910), exceeding the late fusion model (AUC = 0.892), the ViT model (AUC = 0.870), and the Rad model (AUC = 0.792). It also demonstrated superior accuracy (0.848), sensitivity (0.872), and specificity (0.815). Decision curve analysis further confirmed its clinical utility.

**Conclusion:**

Our study demonstrated that integrating radiomics and deep learning contributed to EGFR mutation prediction, providing a non-invasive approach to support personalized treatment decisions in lung adenocarcinoma.

## Introduction

Lung cancer is the most prevalent malignancy globally and the leading cause of cancer-related mortality in China, with non-small cell lung cancer (NSCLC) accounting for 85% of all lung cancer cases (1–3). In recent years, advances in the understanding of genetic alterations have led to the identification of key mutations, including rearrangements of anaplastic lymphoma kinase (ALK), and mutations in Kirsten rat sarcoma virus (KRAS) and epidermal growth factor receptor (EGFR), as critical prognostic factors in lung adenocarcinoma (4). EGFR mutations, primarily exon 19 deletions and the L858R mutation in exon 21, are the most common genetic alterations in lung adenocarcinoma, occurring in approximately 50% of lung adenocarcinoma cases (5). Currently, EGFR detection primarily relies on tumor biopsy sequencing. However, biopsy is an invasive procedure that may increase the risk of cancer metastasis and lead to complications such as bleeding and pneumothorax (6). Additionally, challenges such as inadequate or difficult sample collection, the need for repeated sampling, and the high costs associated with sequencing underscore the limitations of this approach (7, 8). In this context, there is an urgent need to explore low-risk, non-invasive alternatives for predicting EGFR mutations.

High-resolution chest CT is widely used for lung lesion assessment due to its non-invasive nature and ease of operation. Previous studies have commonly utilized CT imaging features, machine learning, or radiomics to predict EGFR mutations (9–12). For example, Giovanni et al. explored radiomics for EGFR mutation prediction (11), while Pinheiro et al. examined its association with imaging phenotypes (9). Traditional radiomics, which relies on predefined features such as texture, shape, and intensity, alongside machine learning for classification, is limited by its dependence on manual feature selection, thereby restricting its ability to fully leverage high-dimensional imaging data for EGFR mutation prediction. In recent years, deep learning has gained significant recognition in medical image analysis, particularly for the non-invasive prediction of clinical outcomes (13–16). Deep learning enables end-to-end image analysis by automatically extracting high-dimensional features through neural networks, fully utilizing raw imaging data to capture complex spatial patterns and nonlinear relationships. For example, Zhao et al. developed a deep learning model using a 3D convolutional neural network (CNN) to predict EGFR mutations (15). Additionally, PET/CT imaging combined with ResNet-based models has been successfully employed to predict EGFR mutations (16).

Although both radiomics and deep learning have shown great success in imaging analysis, the use of a single model still has certain limitations. On one hand, radiomics offers clinically interpretable features, while deep learning models, due to their “black-box” nature, make it difficult to interpret the underlying decision-making processes (17). On the other hand, radiomics is particularly advantageous in small-sample datasets, whereas deep learning requires large-scale data for effective training (18). To overcome these limitations, researchers have increasingly explored the integration of radiomics and deep learning, aiming to leverage the strengths of both approaches (13, 14). The integration of radiomics and deep learning primarily involves two strategies: early fusion (feature-level fusion) and late fusion (decision-level fusion). Early fusion involves extracting features from both radiomics and deep learning models, integrating them at the feature level, and inputting them into a classifier for final prediction. In contrast, late fusion trains radiomics and deep learning models independently, then combines their outputs using methods such as weighted averaging, voting, or other ensemble techniques. Studies have shown that fusion models generally outperform single-model approaches in predictive tasks. Specifically, both Pease et al. and Wang et al. demonstrated that fusion models achieved higher area under the curve (AUC) on multi-center datasets compared to models relying solely on radiomics or deep learning (13, 14).

In summary, our study aimed to compare the performance of various deep learning models and radiomics approaches in predicting EGFR mutation status. Furthermore, we explored two fusion strategies to assess their effectiveness in identifying EGFR mutations. We believe that our findings will contribute to more accurate clinical detection of EGFR mutations, ultimately improving the efficiency and accessibility of precision medicine.

## Methods

### Data collection

**Figure 1:**
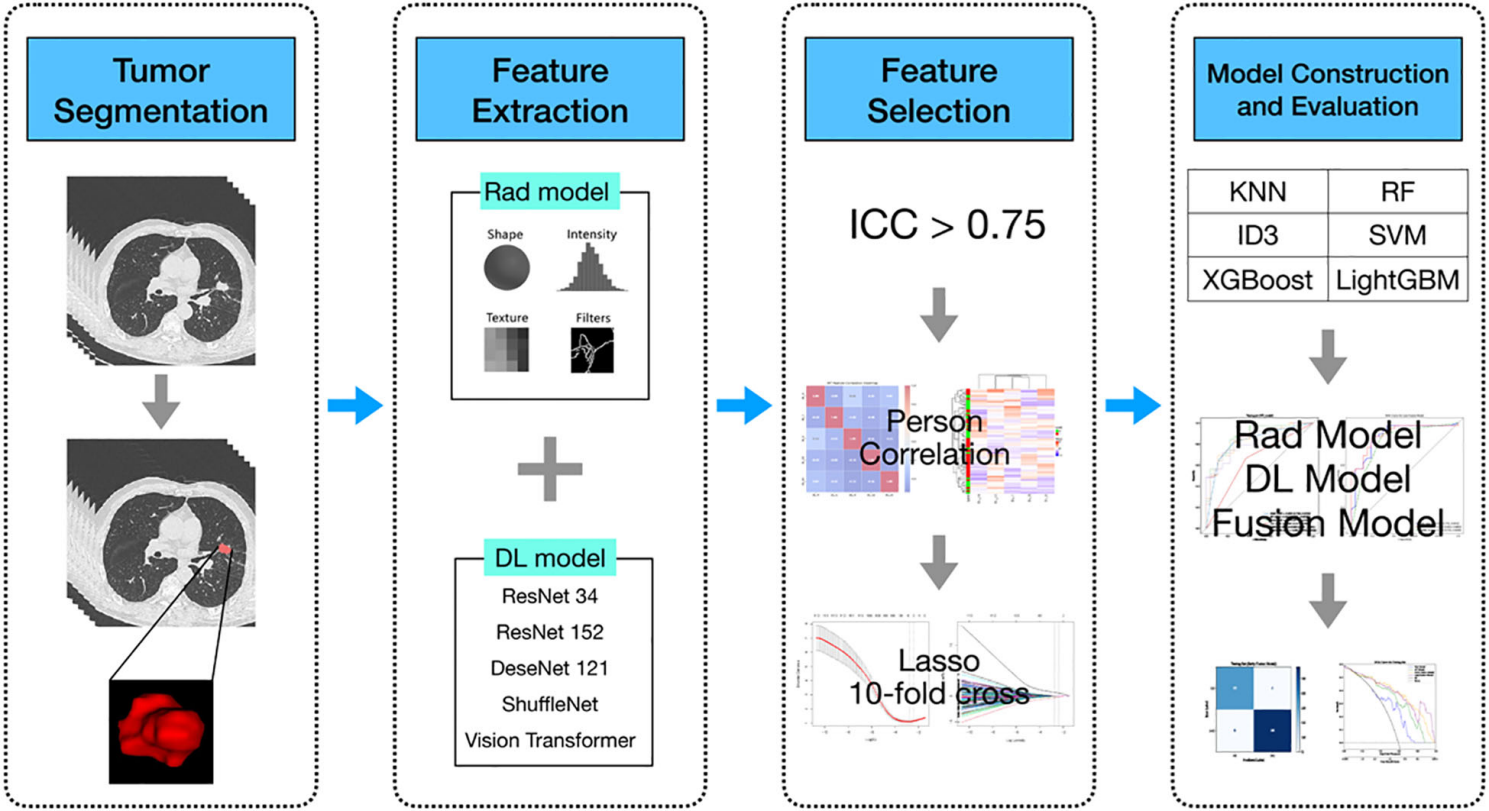
Flowchart of the predictive models. Rad model, Radiomics model; DL model, Deep learning model; AUC, area under the receiver operating characteristics curve; RF, Random Forest; KNN, k-Nearest Neighbors; SVM, Support Vector Machine; ID3, Iterative Dichotomiser 3; XGBoost, eXtremeGradient Boosting; LightGBM, Light Gradient Boosting Machine.

Data from patients diagnosed with lung adenocarcinoma were retrospectively collected at the First People’s Hospital of Yancheng between January and December 2021. The inclusion criteria were as follows (1): histologically confirmed primary lung adenocarcinoma; (2) no prior radiotherapy, chemotherapy, or immunotherapy before surgery; (3) the interval between preoperative CT examination and surgery was within 2 weeks; (4) the patient underwent genetic testing. The exclusion criteria were: (a) *in situ* adenocarcinoma, microinvasive adenocarcinoma, and rare histological variants of lung adenocarcinoma; (b) multiple primary tumors; (c) absence of thin-slice CT images or poor image quality; (d) incomplete clinical, pathological, or genetic data; (e) history of other cancers. Ultimately, a total of 220 patients were included, who were stratified and randomly assigned to the training group (n = 154) and the testing group (n = 66). Of all patients, 122 were diagnosed with EGFR mutations through genetic testing, while the remaining 98 had no detected mutations. The overall workflow of this study is shown in **Figure 1**. This study was approved by the Ethics Committee of the First People’s Hospital of Yancheng (Approval No: 2024-K(YJ)-298) and adheres to the Declaration of Helsinki, with informed consent from participants not being required.

All patients included in the study underwent supine chest CT scanning within two weeks prior to surgery. The lung window (width 1600 HU; level -600 HU) and mediastinal window (width 400 HU; level 40 HU) were fixed, and the chest CT images were resampled to a voxel size of 1mm * 1mm * 1mm using trilinear interpolation to reduce variations in feature values caused by different voxel sizes. All target regions of interest (ROIs) were independently delineated layer by layer by two experienced radiologists (one with 10 years of interpretation experience and the other with 15 years of experience) in a blinded manner using ITK-Snap (www.itksnap.org) software (19). In cases of disagreement, consensus was reached through group discussion. To assess the reliability and consistency of the ROI delineation, all ROIs were re-annotated two months later, and intra-class correlation (ICC) analysis was performed on the data.

### Radiomics model feature extraction

In this study, a total of 1,834 radiomic features, including shape, statistical, and texture features, were extracted using Pyradiomics (configuration in **Supplementary Materials, Section 1**) (20). These features were standardized using Z-scores, and ICC analysis was performed to select features with good consistency (ICC > 0.75). Redundant features were then filtered out based on spearman correlation (Spearman correlation coefficient ≥ 0.9). Subsequently, the Least Absolute Shrinkage and Selection Operator (LASSO) regression with 10-fold cross-validation was applied to select the most predictive subset of features.

### 3D deep learning model feature extraction

Similar to previous studies, we evaluated the performance of several deep learning models using transfer learning, including ResNet34, ResNet152, DenseNet121, ShuffleNet, and Vision Transformer (ViT). The input size for all models was standardized to 64×64×64 voxel cubes of the ROI. Each model was initialized with ImageNet (21) pre-trained weights and trained under a unified data preprocessing pipeline. To ensure fairness and consistency, all models were trained using the same preprocessing steps, with no additional spatial or intensity-based data augmentation applied beyond resampling each manually delineated 3D ROI to 64 × 64 × 64 voxels, and hyperparameters were strictly controlled. Despite differences in network architectures, training parameters were kept consistent across models, including training only the classification head while keeping the backbone weights frozen, the Adam optimizer (initial learning rate = 0.001, weight decay = 1e−4, batch size = 8), and 300 training epochs. The ViT-based deep learning model (ViT model) had specific configurations such as image patch size, frame size, depth, and dimension appropriately adjusted to suit its Transformer architecture, while other training parameters remained consistent with those of the other deep learning models. Specifically, the ViT model was configured with an image patch size of 16 and a frame patch size of 2, resulting in a total of 512 patches per volume. The architecture consisted of 6 Transformer layers (depth = 6), 8 attention heads, and an embedding dimension of 1024, followed by an MLP head with a hidden dimension of 2048. Both dropout and embedding dropout were set to 0.1 (detailed parameters are provided in **Supplementary Materials, Section 2: ViT model settings**). To reduce the risk of overfitting and standardize the feature dimensionality across different architectures, features extracted from the penultimate layer were compressed into 128 dimensions using principal component analysis (PCA).

### Model training and evaluation

To ensure robustness and comparability across different feature types, we applied a unified machine learning framework to both radiomics and deep learning features. Specifically, six commonly used classifiers were evaluated: Random Forest (RF), k-Nearest Neighbors (KNN), Support Vector Machine (SVM), Iterative Dichotomiser 3 (ID3), eXtreme Gradient Boosting (XGBoost), and Light Gradient Boosting Machine (LightGBM). Each classifier was trained using 10-fold stratified cross-validation within the training set. For every classifier, the AUC and its standard deviation across folds were computed. The classifier with the highest mean AUC was selected as the final model for each feature type. These optimal classifiers were then retrained on the training cohort and subsequently evaluated on the testing set. As a result, the SVM classifier combined with radiomics features (Rad model with SVM) and with ViT-derived deep learning features (ViT model with SVM) achieved the best performance and were used in subsequent analyses.

### Construction of the fusion model

Furthermore, we developed early and late fusion models using radiomics and deep learning features. In early fusion, we first applied Z-score normalization to standardize the features. Then, we performed ICC analysis, spearman correlation analysis, and lasso regression for feature selection. The selected features were subsequently used to develop the early fusion model with an SVM classifier. In contrast, late fusion was performed by integrating the prediction outputs from radiomics and deep learning models using various stacked ensemble learning strategies, including RF, SVM, and KNN. SVM was ultimately chosen to develop the final late fusion model. To account for the slight imbalance in EGFR mutation status, the class_weight=balanced parameter was applied to all SVM classifiers. This allowed automatic adjustment of class weights according to class frequencies during training.

### Statistical analysis

The t-test or the Mann-Whitney U test was used to analyze continuous variables, while the chi-square (χ²) test was used to assess categorical variables. The diagnostic efficacy of the models was evaluated using receiver operating characteristic (ROC) curves, AUC, accuracy, and specificity, and other relevant metrics. The DeLong test was applied to compare AUC values. Model calibration was assessed using calibration curves, and decision curve analysis (DCA) was conducted to evaluate the clinical utility of our predictive models.

All data analyses were performed using Python (version 3.11) and R (version 4.4). Radiomics feature extraction was conducted with PyRadiomics (version 3.1.0). Machine learning models, including SVM, were implemented using Scikit-learn, while deep learning models were developed using the PyTorch framework.

## Results

This retrospective study included 220 patients, with their baseline characteristics summarized in **Table 1**. The mean age was 63.5 ± 9.3 years in the training set and 64.35 ± 9.6 years in the testing set, with no significant difference. The proportion of male patients was similarly comparable at 47.4% and 48.5%, respectively. The right upper and left upper lobes were the most frequently affected lesion sites in both cohorts. Tumor staging showed no significant difference (p = 0.249), with stage I being the most common in both sets. The prevalence of EGFR mutations was 54.5% and 57.6% in the training and testing sets, respectively. In addition, a detailed comparison between the EGFR mutant and wild-type groups is provided in **Supplementary Table S1**.

**Table 1:** Baseline characteristics of study sets.

Characteristics	Train set (n = 154)	Test set (n = 66)	*P* value
Age (years)	63.5 ± 9.3	64.35 ± 9.6	0.564
Sex			
Male	73	32	0.999
Female	81	34	
Lesion site			
Right upper	51	19	0.720
Right middle	12	4	
Right lower	31	12	
Left upper	31	19	
Left lower	29	12	
Tumor stage			
I	87	35	0.249
II	13	10	
III	25	6	
IV	29	15	
EGFR			
Yes	84	38	0.789
No	70	28	

EGFR, epidermal growth factor receptor.

After performing feature selection, we identified 19 key radiomics features from the initial set of 1834 features. Additionally, feature selection identified 7, 7, 9, 8, and 5 features for ResNet34, ResNet152, DenseNet121, ShuffleNet, and ViT, respectively. Furthermore, we presented five plots for each model in the **Supplementary Materials**, including Lasso-selected feature plots, feature weight plots, Spearman correlation analysis plots, and hierarchical clustering heatmaps (see **Supplementary Figure S1-Figure S6**). These visualizations offered a deeper understanding of the relationships between the selected features, their weights, and their correlations with each other.

**Figure 2:**
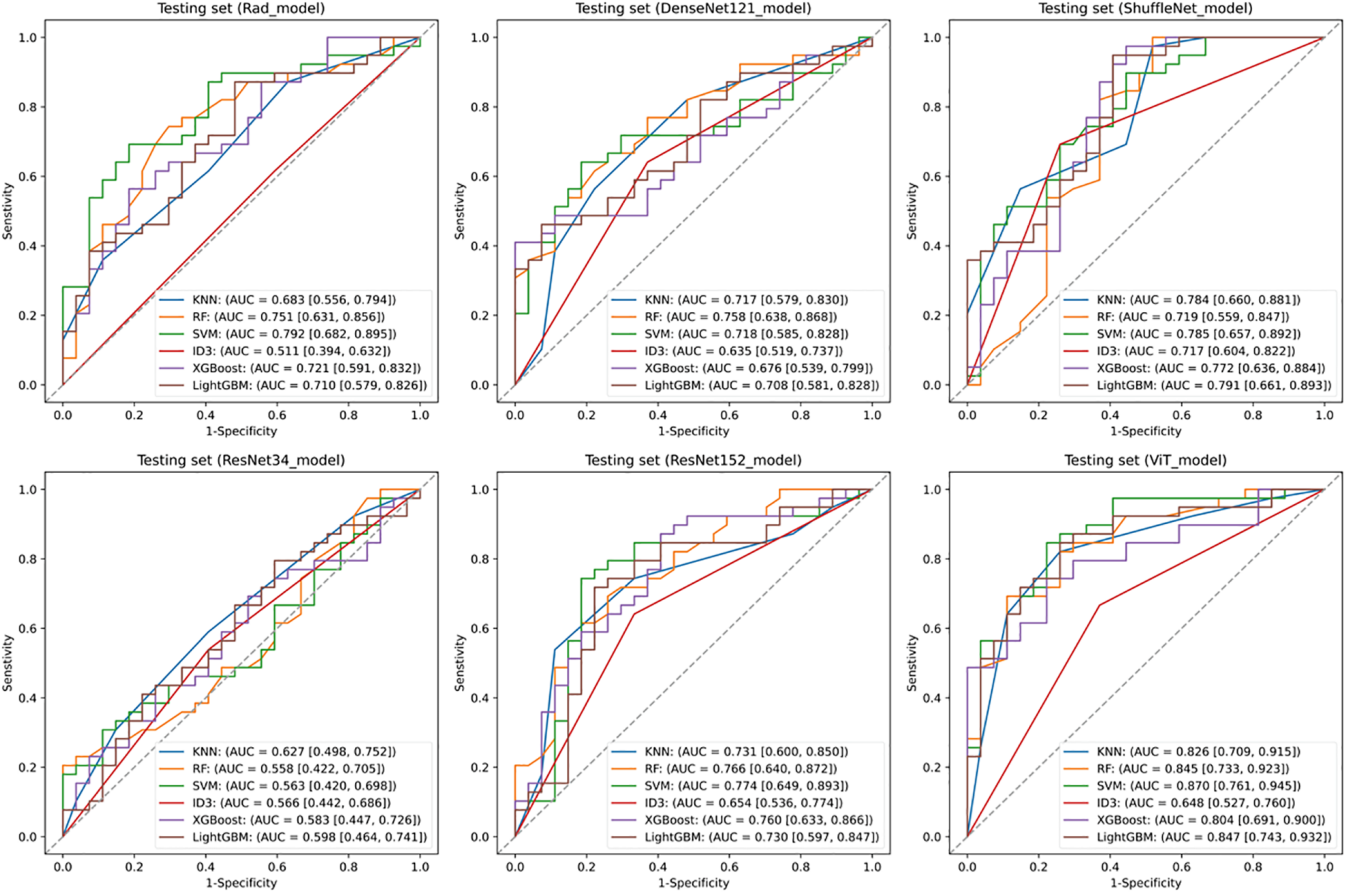
Predictive performance of EGFR. Receiver operating characteristic (ROC) curves of the radiomics model and deep learning models in the testing set. SVM, Support Vector Machine; KNN, k-Nearest Neighbors; ID3, Iterative Dichotomiser 3; RF, Random Forest; XGBoost, eXtreme Gradient Boosting; LightGBM, Light Gradient Boosting Machine.

**Figure 2** illustrates the comparative discriminative performance of radiomics and deep learning models for EGFR mutation status prediction. Radiomics features were extracted using PyRadiomics and used to train RF, KNN, SVM, ID3, XGBoost, and LightGBM classifiers. Among these classifiers, SVM achieved the highest performance on the testing set, with an ROC-AUC of 0.792 (95% CI: 0.682–0.895). For deep learning models, features were extracted from ResNet34, ResNet152, DenseNet121, ShuffleNet, and ViT, and were further classified using the same classifiers. The predictive performance varied considerably among models, with ResNet34 achieving the lowest AUC (< 0.600), whereas ViT combined with an SVM classifier attained the highest AUC of 0.870 (95% CI: 0.761–0.945). Among all deep learning models, ViT demonstrated the best performance, surpassing the optimal radiomics-based model. This finding suggests that deep features extracted by ViT offer superior discriminative ability in predicting EGFR mutation status.

**Figure 3:**
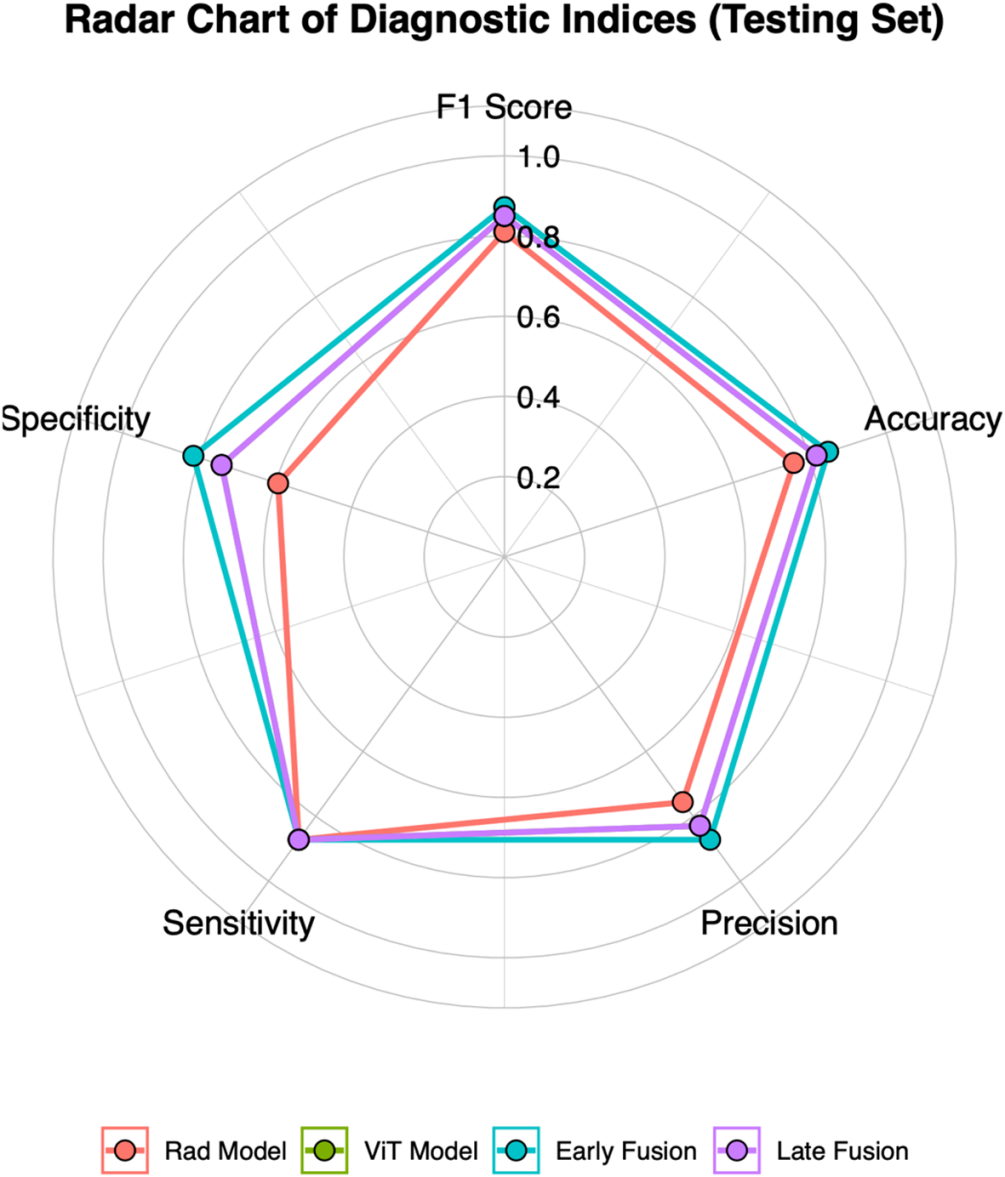
Radar chart of five diagnostic metrics (Accuracy, Sensitivity, Specificity, Precision, and F1 Score) for four models in the testing set.

This study employed early and late fusion strategies to integrate radiomics and deep learning features. In late fusion, we applied ensemble learning methods, including RF, SVM, and KNN, with SVM demonstrating the highest performance (AUC = 0.892, 95% CI: 0.813–0.960) (see **Supplementary Figure S7**). To comprehensively assess the predictive performance of different models, the evaluation results of the Rad model, ViT model, early fusion model, and late fusion model in both the training and testing sets are provided in **Supplementary Tables S2** and **Supplementary Tables S3**, with the corresponding visualizations shown in **Figure 3** and **Supplementary Figure S8**. The reported metrics include AUC with 95% CI, accuracy, sensitivity, specificity, precision, F1 score, and P-values obtained from DeLong’s test. In the training set, the early fusion model demonstrated the highest predictive performance, achieving an AUC of 0.965 (95% CI: 0.934–0.989), and was used as the reference. The late fusion model also exhibited strong discriminative ability, with an AUC of 0.945 (95% CI: 0.908–0.976). The ViT model achieved an AUC of 0.895 (95% CI: 0.845–0.941), outperforming the Rad model (AUC = 0.877, 95% CI: 0.824–0.926). Both models showed statistically significant differences when compared with the early fusion model (P < 0.05, DeLong’s test). In addition to AUC, the early fusion model achieved the highest accuracy (0.909), sensitivity (0.916), and specificity (0.901). Moreover, the optimal threshold for the early fusion model in the training set was 0.464, determined using the maximum Youden index, which yielded a sensitivity of 95.2% and a specificity of 88.7%. In the testing set, the early fusion model maintained the best discriminative performance, with an AUC of 0.910 (95% CI: 0.822–0.970), serving as the reference. The late fusion model followed with an AUC of 0.892 (95% CI: 0.813–0.960), while the ViT model (AUC = 0.870, 95% CI: 0.761–0.945) outperformed the Rad model (AUC = 0.792, 95% CI: 0.682–0.895). Comparisons with the early fusion model revealed statistically significant differences for both models (P < 0.05, DeLong’s test). In addition, the optimal threshold for the early fusion model in the testing set was 0.519, corresponding to a sensitivity of 87.2% and specificity of 81.5%. The model also achieved the highest accuracy (0.848) among all models in the testing set, further highlighting its predictive superiority.

**Figure 4:**
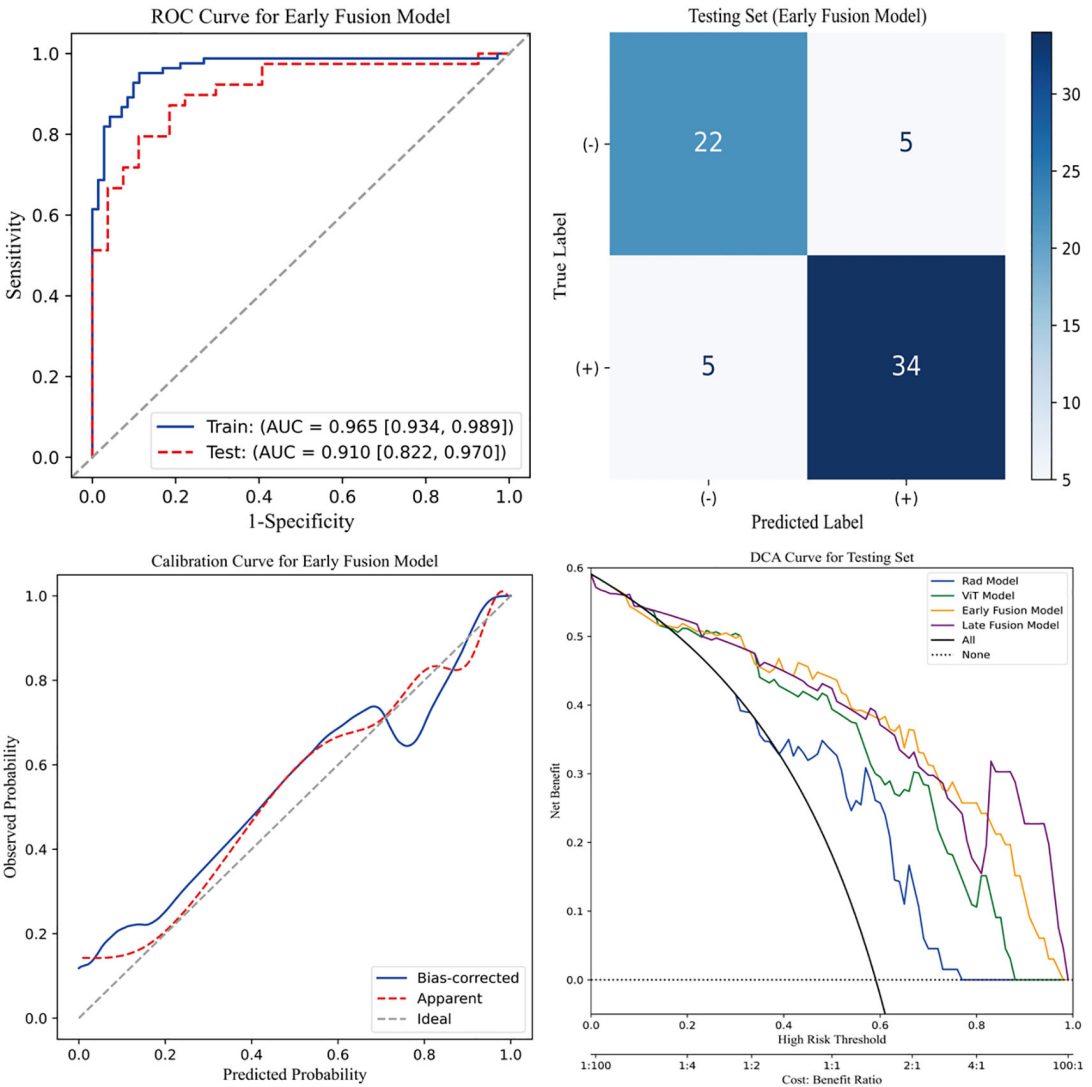
Evaluation of the early fusion model. The top-left panel depicts the Receiver Operating Characteristic (ROC) curve of the early fusion model, while the top-right panel presents the corresponding confusion matrix for the test cohort. The bottom-left panel illustrates the calibration curve of the early fusion model, indicating good model calibration. Furthermore, the bottom-right panel displays the Decision Curve Analysis (DCA), revealing that the early fusion model achieves the highest clinical net benefit.

**Figure 4** presents four key visualizations for assessing the performance of the early fusion model. The ROC curve illustrates the model’s discriminative ability, with the AUC reflecting its overall classification performance. The confusion matrix visually represents classification outcomes, detailing the distribution of true positives, true negatives, false positives, and false negatives. To assess the model’s calibration, a calibration curve is included, demonstrating the agreement between predicted probabilities and actual outcomes. Additionally, DCA is performed to evaluate the model’s clinical utility by quantifying the net benefit across a range of threshold probabilities. The clinical net benefit of the predictive models emerged only when the threshold probability exceeded approximately 0.2. Below this threshold, the net benefit was comparable to the treat-all strategy, indicating limited additional value for decision-making. Within the 0.2 to 0.8 range, the early fusion model consistently demonstrated the highest net benefit, outperforming both reference strategies and other models. Collectively, these analyses offer a thorough assessment of the early fusion model’s predictive performance, reliability, and potential clinical applicability.

## Discussion

EGFR mutation status is a critical determinant of personalized treatment strategies in lung adenocarcinoma, directly affecting the selection of targeted therapies and prognostic evaluation (5, 22). In our study, radiomics and deep learning approaches were integrated to construct and compare multiple predictive models for EGFR mutation status. Among the individual models, the ViT model exhibited the highest predictive performance. Notably, the fusion models, particularly the early fusion approach, achieved further improvements in predictive accuracy. In the testing set, the early fusion model achieved an AUC of 0.910, outperforming both the radiomics-based and standalone deep learning models, underscoring the advantages of multimodal feature integration for EGFR mutation prediction. Furthermore, DCA validated the model’s potential clinical utility, reinforcing its significance in non-invasive biomarker assessment.

EGFR mutation is a crucial biomarker for guiding personalized treatment in lung adenocarcinoma, and its accurate prediction plays a vital role in treatment decision-making and prognosis assessment. With the increasing application of radiomics in medicine, growing evidence suggests that non-invasive CT-based radiomics models outperform traditional imaging features in predictive accuracy and clinical utility (23–25). Dong et al. reported that a radiomics-based nomogram achieved an AUC of 0.798 (95% CI: 0.664–0.931) in the validation cohort for EGFR mutation prediction (26). Similarly, Dang et al. demonstrated that radiomics (AUC = 0.703) outperformed clinical features (AUC = 0.284) in predicting EGFR mutation status (23). Furthermore, a meta-analysis of 28 radiomics-based studies reported a pooled AUC of 0.800 (95% CI: 0.757–0.845), indicating a moderate-to-high predictive accuracy and a significant improvement over clinical features (24). Despite the promising performance of radiomics models in EGFR mutation prediction, the rapid advancement of deep learning has introduced new breakthroughs, particularly by automating feature extraction and capturing complex patterns, further enhancing predictive accuracy. Yin et al. constructed a deep learning model using ResNet, achieving an AUC of 0.84 (95% CI: 0.75–0.90) in EGFR mutation prediction (27). Similarly, another study reported that a CNN-based model (AUC = 0.7802) outperformed radiomics (AUC = 0.7038) in predictive accuracy (6). Moreover, a joint study conducted by researchers from the United Kingdom and India demonstrated that a 3D U-Net-based deep learning model (AUC = 0.82, 95% CI: 0.81–0.83) exhibited superior performance compared to radiomics (AUC = 0.72, 95% CI: 0.69–0.75) (28). Similar to most previous studies, our study found that deep learning models generally outperformed radiomics models in predicting EGFR mutation status. Notably, among all deep learning architectures, ViT combined with an SVM classifier achieved the highest AUC (0.870, 95% CI: 0.761–0.945), demonstrating superior performance. This model not only outperformed all other deep learning models but also surpassed the best-performing radiomics model.

The superior performance of ViT combined with an SVM classifier can be attributed to its ability to model global dependencies through self-attention mechanisms, enabling it to capture subtle and spatially distributed imaging patterns associated with EGFR mutation (29). Compared to conventional CNNs, ViT’s long-range feature extraction enhances its capability to identify mutation-related characteristics beyond localized regions (29, 30). Additionally, the integration of SVM as a classifier may have contributed to improved generalization, reducing the risk of overfitting. These factors collectively explain why ViT model with SVM outperformed both other deep learning models and radiomics-based approaches in our study.

Interestingly, we observed that ResNet34 performed worse than the radiomics-based model, a finding that contrasts with the general trend of deep learning models outperforming traditional feature-based approaches. This result suggests that not all deep learning architectures are equally effective for EGFR mutation prediction, and several factors may explain ResNet34’s inferior performance. One possible reason is its relatively shallow architecture, which may limit its ability to extract high-level imaging features essential for distinguishing EGFR mutation status (31). Furthermore, deep CNN models typically require large-scale training data to fully optimize their parameters, whereas our dataset may have been insufficient for ResNet34 to generalize effectively (32). In contrast, radiomics models rely on predefined feature extraction methods that remain relatively stable even with limited sample sizes, potentially contributing to their superior performance in this setting.

Furthermore, consistent with previous studies (6, 14, 33), our study found that fusion models outperformed single-feature models in predicting EGFR mutation status, highlighting the complementary nature of radiomics and deep learning-derived features. The superior performance of fusion models can be attributed to the complementary nature of radiomics and deep learning-derived features, which capture distinct yet synergistic aspects of tumor characteristics (33). This synergistic advantage has also been demonstrated in recent studies that integrated deep learning architectures with radiomics for improved prediction and clinical applicability across different disease contexts (34, 35). Radiomics extracts predefined morphological and textural attributes, while deep learning autonomously learns high-level representations, enabling a more comprehensive analysis. This synergy enhances predictive performance, as evidenced by the superior AUC achieved by the fusion models. In addition to AUC, precision and F1-score provide further insights into model performance. The high precision of the early fusion model suggests a low false-positive rate, which is clinically meaningful in reducing unnecessary EGFR testing. The superior F1-score also indicates a favorable balance between sensitivity and precision, reflecting the robustness of this model in classifying EGFR mutation status. These results support the effectiveness of early fusion strategies, which leverage the strengths of both handcrafted and deep-learned features to improve classification accuracy.

Although our study has provided valuable insights, several limitations should be acknowledged. First, as a retrospective study with a limited sample size from a single center, our model lacks external validation, which may restrict its generalizability for clinical applications. Future prospective studies with larger, multi-center cohorts are necessary to ensure the robustness of our findings and enhance the model’s applicability across diverse clinical settings. Second, due to the lack of significant differences in baseline clinical variables between the EGFR mutant and wild-type groups in our cohort, we focused on imaging data to evaluate the independent predictive value of radiomics and deep learning. Nevertheless, incorporating additional clinical variables, such as smoking history, may further enhance model performance and should be considered in future studies. Third, this study was conducted exclusively in an Asian population, whereas EGFR mutation prevalence varies across different ethnic groups (36). This limitation may affect the generalizability of our findings. Further research with multi-ethnic cohorts is needed to evaluate the applicability of radiomics-based models across diverse populations. Finally, although our fusion models demonstrated favorable predictive performance, the lack of inherent interpretability in the SVM classifier limits their clinical transparency. Future research should incorporate explainable AI techniques or feature attribution methods to enhance model interpretability, which is critical for clinical decision-making. In summary, despite these limitations, our study highlights the potential of integrating radiomics and deep learning for EGFR mutation prediction. Future research should aim to validate our findings in multi-center cohorts, incorporate multimodal clinical data, and extend applicability to diverse populations to enhance the clinical utility of radiomics-based models.

## Conclusion

In this study, we developed a predictive model for EGFR mutation status in lung adenocarcinoma using CT-based radiomics and deep learning. Compared to traditional radiomics models and individual deep learning architectures, our fusion model demonstrated significantly improved predictive performance, highlighting the complementary strengths of handcrafted and deep-learned features. This non-invasive approach provides a valuable alternative to biopsy-based genetic testing, mitigating the risks and limitations associated with invasive procedures while facilitating the identification of EGFR mutation status in certain lung adenocarcinoma patients. It offers a potential tool for improving early diagnosis and treatment stratification.

## Data Availability

The raw data supporting the conclusions of this article will be made available by the authors, without undue reservation.
