# Supplementary material for: CT-based radiomics and deep learning to predict EGFR mutation status in lung adenocarcinoma
Source: Front Oncol. 2025 Oct 2;15:1597548. doi: 10.3389/fonc.2025.1597548 (PMC12527853; doi:10.3389/fonc.2025.1597548)
Supplement: Supplementary file 1 [file DataSheet1.docx]

**CT-Based Radiomics and Deep Learning to Predict EGFR Mutation Status in NSCLC**

**1. Configuration file for Pyradiomics**

imageType:

Original: {}

LoG:

sigma: [1.0, 2.0, 3.0] # If you include sigma values >5, remember to also increase the padDistance.

Wavelet: {}

LBP3D: {}

Exponential: {}

Square: {}

SquareRoot: {}

Logarithm: {}

Gradient: {}

featureClass:

shape:

firstorder:

glcm: # Disable SumAverage by specifying all other GLCM features available

- 'Autocorrelation'

- 'JointAverage'

- 'ClusterProminence'

- 'ClusterShade'

- 'ClusterTendency'

- 'Contrast'

- 'Correlation'

- 'DifferenceAverage'

- 'DifferenceEntropy'

- 'DifferenceVariance'

- 'JointEnergy'

- 'JointEntropy'

- 'Imc1'

- 'Imc2'

- 'Idm'

- 'Idmn'

- 'Id'

- 'Idn'

- 'InverseVariance'

- 'MaximumProbability'

- 'SumEntropy'

- 'SumSquares'

glrlm:

glszm:

gldm:

ngtdm:

setting:

# Normalization:

# most likely not needed, CT gray values reflect absolute world values (HU) and should be comparable between scanners.

# If analyzing using different scanners / vendors, check if the extracted features are correlated to the scanner used.

# If so, consider enabling normalization by uncommenting settings below:

#normalize: true

#normalizeScale: 500 # This allows you to use more or less the same bin width.

# Resampling:

# Usual spacing for CT is often close to 1 or 2 mm, if very large slice thickness is used,

# increase the resampled spacing.

# On a side note: increasing the resampled spacing forces PyRadiomics to look at more coarse textures, which may or

# may not increase accuracy and stability of your extracted features.

interpolator: 'sitkBSpline'

resampledPixelSpacing: [1, 1, 1]

padDistance: 10 # Extra padding for large sigma valued LoG filtered images

# Mask validation:

# correctMask and geometryTolerance are not needed, as both image and mask are resampled, if you expect very small

# masks, consider to enable a size constraint by uncommenting settings below:

#minimumROIDimensions: 2

#minimumROISize: 50

# Image discretization:5

# The ideal number of bins is somewhere in the order of 16-128 bins. A possible way to define a good binwidt is to

# extract firstorder:Range from the dataset to analyse, and choose a binwidth so, that range/binwidth remains approximately

# in this range of bins.

binCount: 64

# first order specific settings:

# This amount is added to the gray level intensity in features Energy, Total Energy and RMS, this is to prevent negative values.

# If using CT data, or data normalized with mean 0, consider setting this parameter to a fixed value (e.g. 2000) that ensures non-

negative numbers in the image.

voxelArrayShift: 1000

# Misc:

# default label value. Labels can also be defined in the call to featureextractor.execute, as a commandline argument,

# or in a column "Label" in the input csv (batchprocessing)

# label: 1

**2. ViT model settings**

image_size: 64

frames: 64

image_patch_size: 16

frame_patch_size: 2

dim: 1024

depth: 6

heads: 8

mlp_dim: 2048

dropout: 0.1

emb_dropout: 0.1

batch_size: 4

epochs: 300

optimizer: Adam

initial_learning_rate: 0.001

weight_decay: 1e-4

**3. Supplementary Figures**


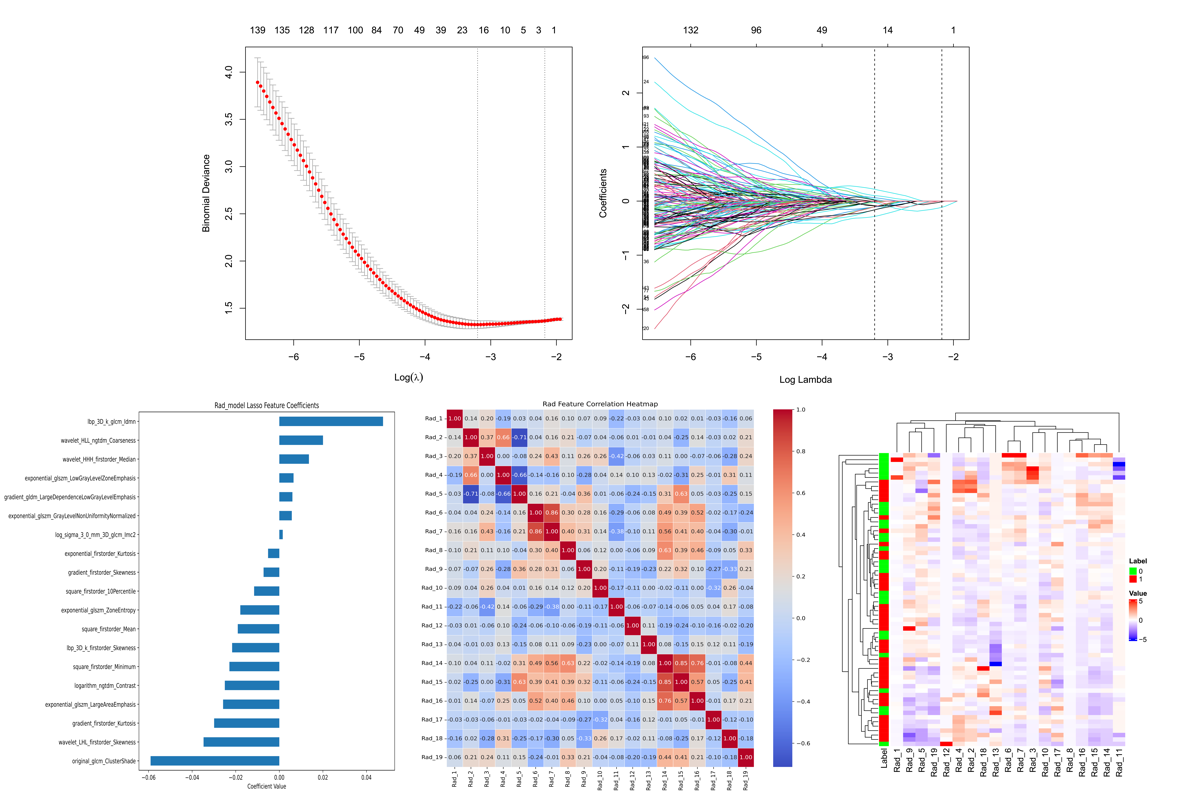


Figure S1: Lasso regression results for Rad features, including the lambda selection curve (top left), coefficient shrinkage path (top right), feature importance bar plot (bottom left), feature correlation heatmap (bottom center), and hierarchical clustering heatmap (bottom right).


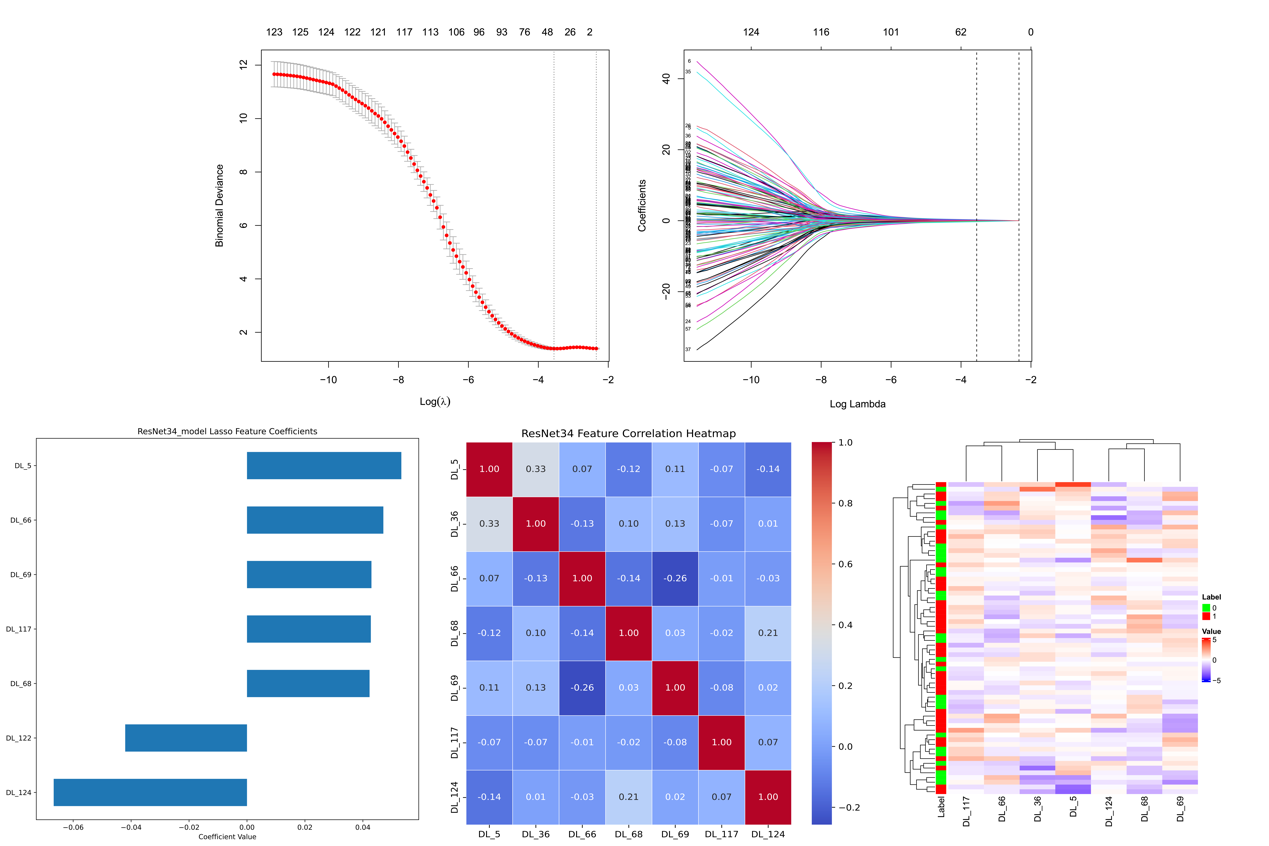


Figure S2: Lasso regression results for ResNet34 features, including the lambda selection curve (top left), coefficient shrinkage path (top right), feature importance bar plot (bottom left), feature correlation heatmap (bottom center), and hierarchical clustering heatmap (bottom right).


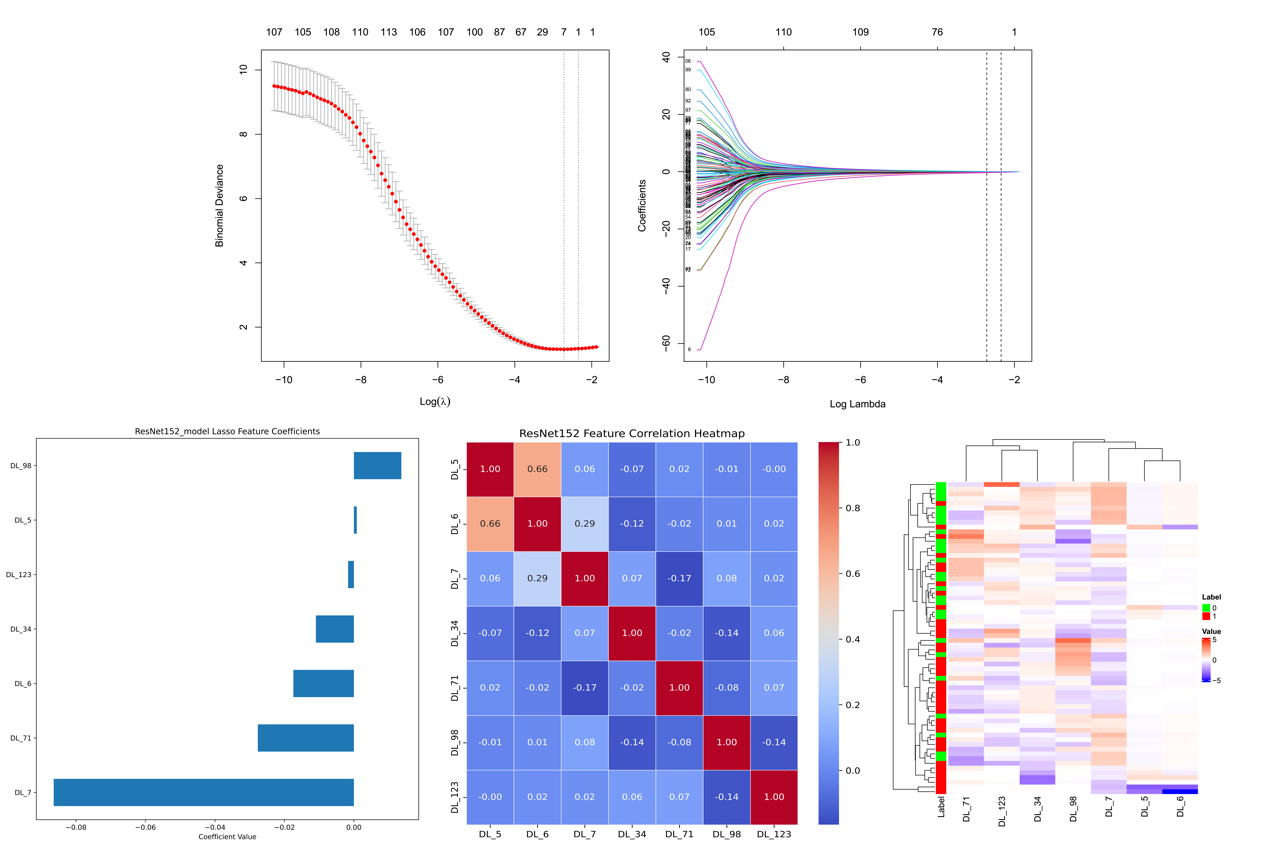


Figure S3: Lasso regression results for ResNet152 features, including the lambda selection curve (top left), coefficient shrinkage path (top right), feature importance bar plot (bottom left), feature correlation heatmap (bottom center), and hierarchical clustering heatmap (bottom right).


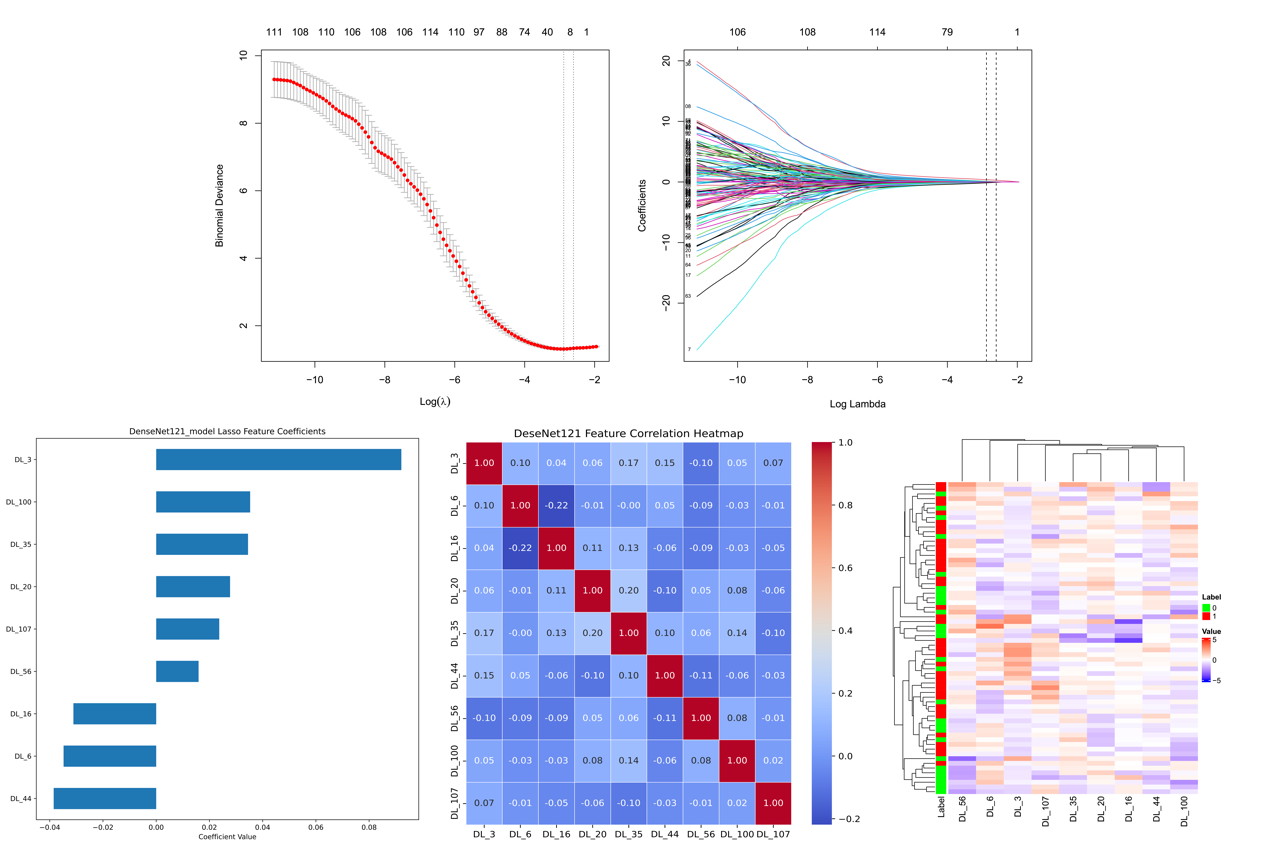
Figure S4: Lasso regression results for DenseNet121 features, including the lambda selection curve (top left), coefficient shrinkage path (top right), feature importance bar plot (bottom left), feature correlation heatmap (bottom center), and hierarchical clustering heatmap (bottom right).


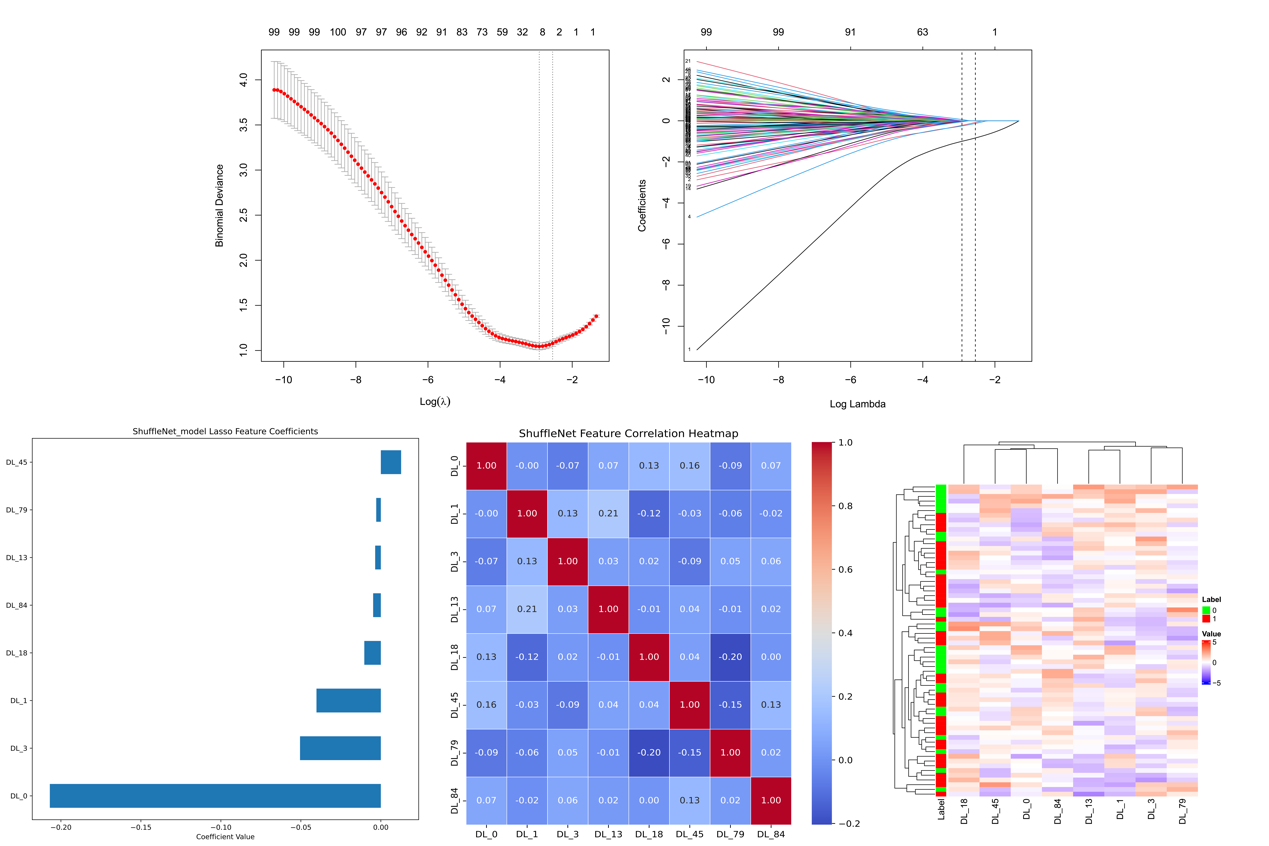
Figure S5: Lasso regression results for ShuffleNet features, including the lambda selection curve (top left), coefficient shrinkage path (top right), feature importance bar plot (bottom left), feature correlation heatmap (bottom center), and hierarchical clustering heatmap (bottom right).


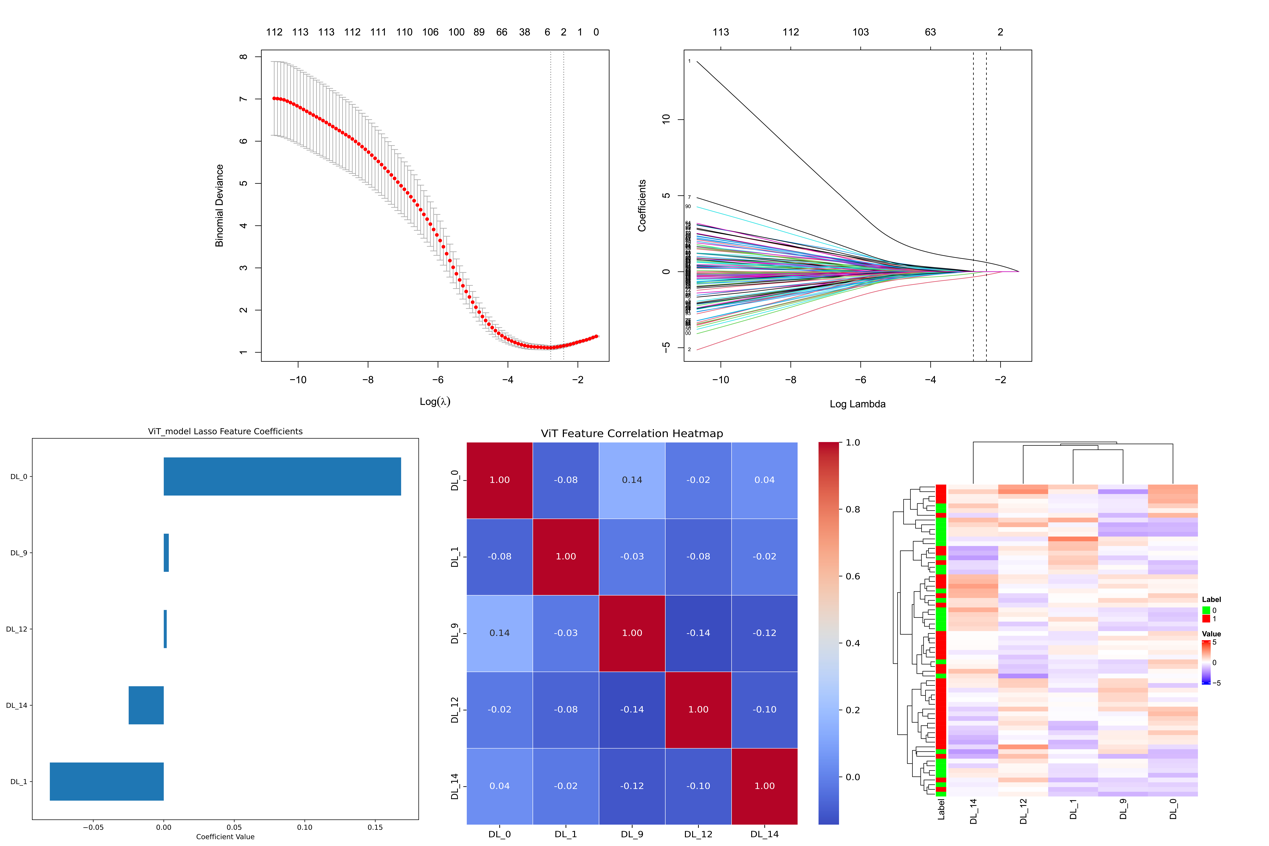
Figure S6: Lasso regression results for ViT features, including the lambda selection curve (top left), coefficient shrinkage path (top right), feature importance bar plot (bottom left), feature correlation heatmap (bottom center), and hierarchical clustering heatmap (bottom right).


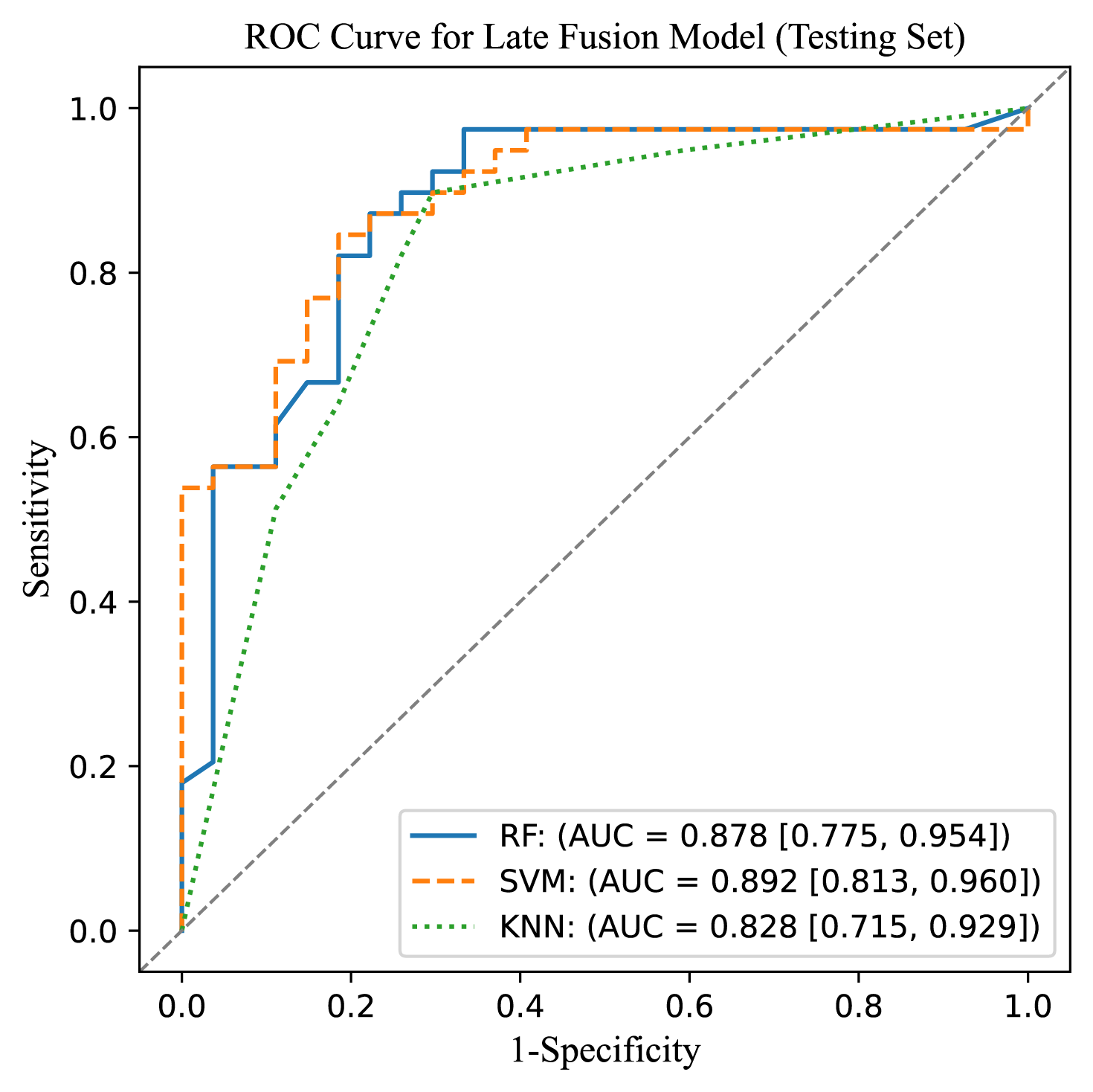


Figure S7: ROC curves of the late fusion model in the testing set using three ensemble learning methods, including Random Forest (RF), Support Vector Machine (SVM), and k-Nearest Neighbors (KNN), with their corresponding AUC values.


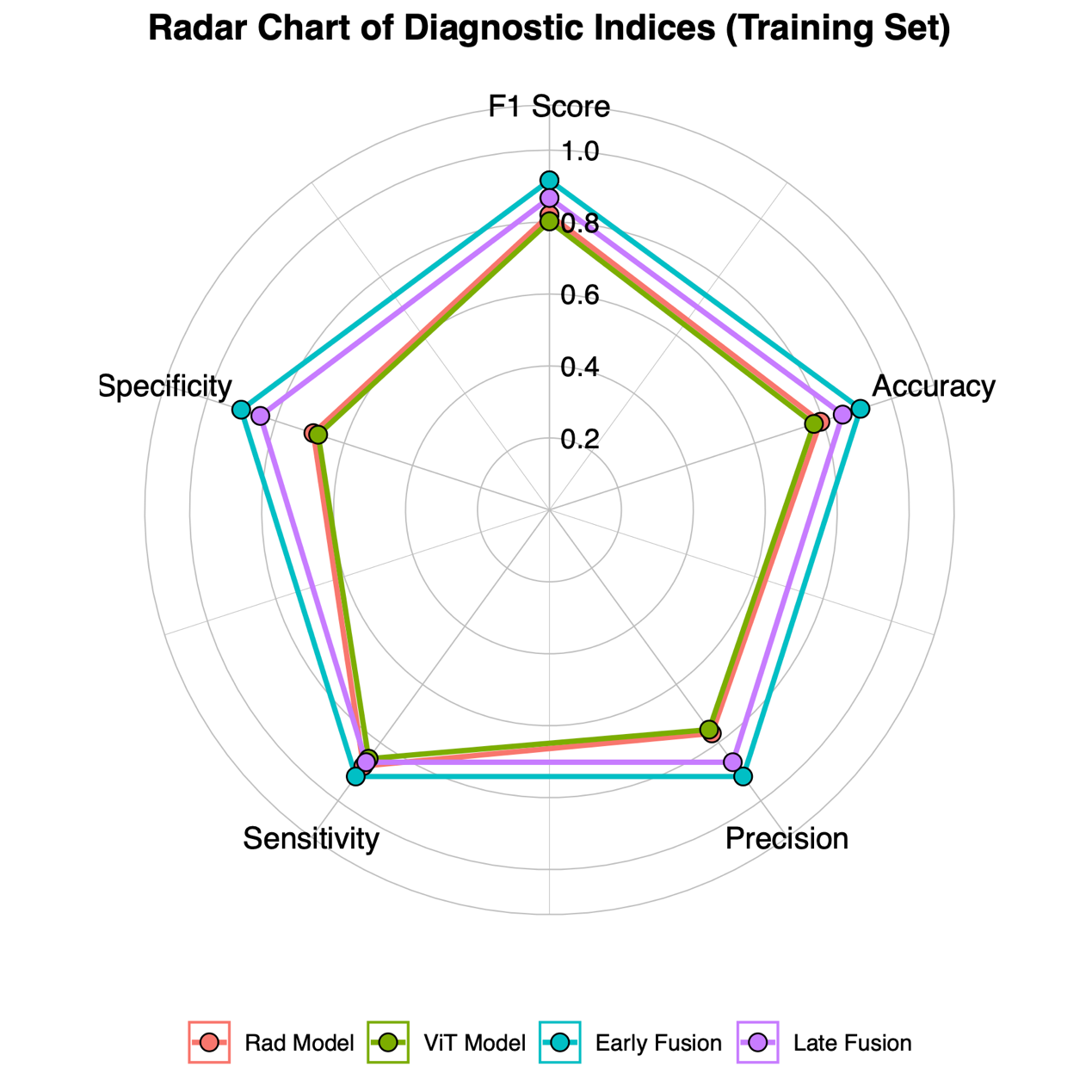
Figure S8: Radar chart of five diagnostic metrics (Accuracy, Sensitivity, Specificity, Precision, and F1 Score) for four models in the training set.

**4. Supplementary Tables**

Table S1: Baseline characteristics of study sets.

| Characteristics | EGFR wild type (n = 98) | EGFR mutant (n = 122) | p value |
| --- | --- | --- | --- |
| Age (years) | 64.7 ± 10.7 | 64.0 ± 8.8 | 0.207 |
| Sex |  |  |  |
| Male | 54 | 51 | 0.068 |
| Female | 44 | 71 |  |
| Lesion site |  |  |  |
| Right upper | 28 | 42 | 0.176 |
| Right middle | 8 | 8 |  |
| Right lower | 24 | 19 |  |
| Left upper | 25 | 25 |  |
| Left lower | 13 | 28 |  |
| Tumor stage |  |  |  |
| I | 39 | 53 | 0.345 |
| II | 14 | 9 |  |
| III | 15 | 16 |  |
| IV | 30 | 44 |  |

Abbreviations: EGFR: epidermal growth factor receptor.

Table S2: Comparison of the AUC values among models in the training and testing sets.

| Models | AUC (95%CI) | *P*-value |
| --- | --- | --- |
| Train |  |  |
| Rad_model | 0.877 (0.824 - 0.926) | **0.001** |
| ViT_model | 0.895 (0.845 - 0.941) | **0.001** |
| Early fusion | 0.965 (0.934 - 0.989) | Reference |
| Late fusion | 0.945 (0.908 - 0.976) | 0.816 |
| Test |  |  |
| Rad_model | 0.792 (0.682 - 0.895) | **0.001** |
| ViT_model | 0.870 (0.761 - 0.945) | **0.021** |
| Early fusion | 0.910 (0.822 - 0.970) | Reference |
| Late fusion | 0.892 (0.813 - 0.960) | 0.323 |

Abbreviations: AUC: Area Under Curve. Rad model: Radiomics model. ViT model: Vision Transformer model.

Table S3: Comparison of the five diagnostic metrics among models in the training and testing sets.

| Models | Accuracy | Sensitivity | Specificity | Precision | F1 Score |
| --- | --- | --- | --- | --- | --- |
| Train |  |  |  |  |  |
| Rad_model | 0.792 (0.721 - 0.857) | 0.880 (0.811 - 0.943) | 0.690 (0.577 - 0.789) | 0.768 (0.680 - 0.848) | 0.820 (0.758 - 0.877) |
| ViT_model | 0.773 (0.708 - 0.838) | 0.855 (0.773 - 0.926) | 0.676 (0.562 - 0.783) | 0.755 (0.670 - 0.837) | 0.802 (0.737 - 0.862) |
| Early fusion | 0.909 (0.864 - 0.948) | 0.916 (0.852 - 0.974) | 0.901 (0.828 - 0.968) | 0.916 (0.854 - 0.974) | 0.916 (0.865 - 0.955) |
| Late fusion | 0.857 (0.799 - 0.909) | 0.867 (0.792 - 0.933) | 0.845 (0.761 - 0.932) | 0.867 (0.793 - 0.938) | 0.867 (0.808 - 0.918) |
| Test |  |  |  |  |  |
| Rad_model | 0.758 (0.652 - 0.864) | 0.872 (0.750 - 0.971) | 0.593 (0.407 - 0.774) | 0.756 (0.625 - 0.880) | 0.810 (0.704 - 0.887) |
| ViT_model | 0.818 (0.712 - 0.909) | 0.872 (0.757 - 0.973) | 0.741 (0.555 - 0.897) | 0.829 (0.714 - 0.944) | 0.850 (0.753 - 0.921) |
| Early fusion | 0.848 (0.772 - 0.924) | 0.872 (0.763 - 0.969) | 0.815 (0.654 - 0.957) | 0.872 (0.762 - 0.971) | 0.872 (0.783 - 0.941) |
| Late fusion | 0.818 (0.712 - 0.909) | 0.872 (0.757 - 0.971) | 0.741 (0.560 - 0.893) | 0.829 (0.714 - 0.929) | 0.850 (0.750 - 0.923) |

Abbreviations: Rad model: Radiomics model. ViT model: Vision Transformer model.
